# Postural orthostatic tachycardia syndrome in post-COVID-19 long-hauler patients is associated with platelet storage pool deficiency

**DOI:** 10.3389/fmed.2025.1560120

**Published:** 2025-09-11

**Authors:** William T. Gunning, Saira Khan, John W. Spatafore, Beverly L. Karabin, Blair P. Grubb

**Affiliations:** ^1^Department of Pathology, University of Toledo Medical Center, Toledo, OH, United States; ^2^College of Medicine and Life Sciences, University of Toledo Medical Center, Toledo, OH, United States; ^3^Department of Medicine, University of Toledo Medical Center, Toledo, OH, United States

**Keywords:** postural orthostatic tachycardia syndrome (POTS), platelets, storage pool deficiency, COVID-19 long haulers, post-acute sequela of COVID-19 (PASC)

## Abstract

**Introduction:**

Postural orthostatic tachycardia syndrome (POTS), a type of dysautonomia, has been an enigma to many healthcare providers. As many as 80% of coronavirus disease 2019 (COVID-19) long-hauler patients meet the diagnostic criteria for POTS, highlighting awareness of this debilitating multisystem disorder. The etiology of POTS has not been entirely defined, but researchers have speculated that an immunological stressor such as a viral infection might be an initiating event. Prior to the pandemic, we reported that POTS patients have a bleeding diathesis with platelet dense granule storage pool deficiency (δ-SPD).

**Methods:**

This report presents a prospective case–control study (*n* = 252) involving four cohorts, comprising two groups of POTS patients and two groups of healthy controls, to evaluate abnormal bleeding and patient demographics. We compared POTS patients and controls that were naïve to the severe acute respiratory syndrome coronavirus 2 (SARS-CoV-2) virus with subjects that had been infected and subsequently developed POTS or who recovered healthy. Questionnaires were employed to assess bleeding tendencies and the severity of clinical symptoms commonly reported with POTS. We utilized electron microscopy to assess platelet dense granules and enzyme-linked immunosorbent assay (ELISA) to assess COVID-19 and Epstein–Barr viral titers.

**Results:**

The most common bleeding symptom was easy bruising in POTS patients naïve to COVID-19 (79.7%) and POTS post-COVID-19 patients (90.5%). Both groups had δ-SPD with means of 2.52 ± 0.9 and 2.44 ± 0.9 DG/PL, respectively, in contrast to a mean of 4.33 ± 0.6 DG/PL for controls naïve to SARS-CoV-2 infection and 4.19 ± 1.0 DG/PL for controls recovered from the virus (*p* < 0.001).

**Discussion:**

We found that the results between the two POTS groups have no statistically significant difference. Our results identify an additional comorbidity (δ-SPD) in COVID-19 “long haulers”/post-acute sequela of COVID-19 (PASC) patients, frequently seen in POTS, that could explain several disparate symptoms often a??ecting the severity of the condition.

## 1 Introduction

Postural orthostatic tachycardia syndrome (POTS) is one of the most common forms of dysautonomia. It is a complex disorder with a myriad of overlapping and sometimes disparate clinical symptoms. The disorder did not have a specific International Classification of Diseases (ICD) diagnostic code assigned by the Centers for Disease Control (CDC) until October 2022 (ICD-10 G90.A) (1).

The definition of postural orthostatic tachycardia syndrome in our clinic is the presence of symptoms of orthostatic intolerance associated with an increased heart rate of 30 beats per minute (BPM) from the basal rate or a rate that exceeds 120 BPM that occurs within the first 10 min of standing or upright tilt (2–4). The disorder is an abnormal physiological state commonly caused by the inability of the peripheral vasculature to maintain adequate resistance in response to orthostatic stress, resulting in excessive pooling of blood in the more dependent areas of the body (5–7). This functional decline in circulatory volume causes a compensatory increase in heart rate and myocardial contractility. In severe cases, the peripheral vasculature resistance is unable to compensate fully, resulting in a reduction in effective circulation and varying degrees of cerebral hypoperfusion. A decrease in arterial blood pressure below the level of cerebral autoregulation due to venous pooling may result in various symptoms, including dizziness, light-headedness, near syncope, and ultimately syncope (2, 8–34). Postural orthostatic tachycardia syndrome has now become more prominently known since the coronavirus disease 2019 (COVID-19) pandemic, as many severe acute respiratory syndrome coronavirus 2 (SARS-CoV-2) patients with lingering effects of the viral infection (COVID-19 “long haulers”/post-acute sequela of COVID-19 (PASC) patients) present with dysautonomia and similar multiple clinical morbidities that are seen in POTS patients. This has led to POTS being a recognized complication of PASC. Clinicians with interests in dysautonomia have reported that up to 80% of PASC patients meet POTS diagnostic criteria (19–21). Regardless, a majority of POTS patients have not been documented or incorrectly documented, and awareness has increased identification of the condition. A recent publication reports that the incidence rate of POTS has risen from 1.42 cases per 1,000,000 prior to the pandemic to 22.66 cases per 1,000,000 post-COVID-19 (22). The rise in incidence might also be related to the recently established CDC ICD code, as mentioned above. There are also many reports that vaccination against the SARS-CoV-2 virus may induce the development of POTS (23–25). Long-hauler patients experience the same debilitating symptoms as POTS patients, leading efforts such as the Researching COVID to Enhance Recovery (RECOVER) clinical trial to include an AUTONOMIC arm (26).

A direct link to the development of POTS as a sequela of COVID-19 infection in PASC gives great credence to the idea that the etiology of POTS involves an immunologic stressor such as a preceding viral illness. Viral infections induce the innate immune system to respond, ultimately inducing the adaptive immune system to produce antibodies that attack the infectious agent. The blood platelet appears to be an important mediator of both the innate and adaptive immune system (35–37). The platelet is also the primary storage site of serotonin in the peripheral circulation, with up to 99% stored in platelet dense granules (38, 39). Insufficient peripheral serotonin may create conditions that lead to hypotension and, as stated above, could explain many of the clinical symptoms commonly reported by POTS patients. We have previously published a study establishing that a majority of POTS patients are deficient in the number of platelet dense granules and have lower, but still normal, concentrations of serotonin compared to controls (11). We also reported in that study that POTS patients had significant bleeding symptoms, including histories of easy bruising, epistaxis, and, for women, abnormal menstrual bleeding (11). A deficiency of platelet dense granules is known to result in mucocutaneous bleeding symptoms (40–42).

The purpose of this study was to (1) validate our previous study that a majority of individuals diagnosed with POTS have platelet dense (delta) granule storage pool deficiency (δ-SPD) and (2) to compare clinical symptoms and platelet dense granule numbers in patients diagnosed with POTS prior to the pandemic (naïve POTS) with PASC patients diagnosed with POTS. Our working hypothesis, according to the current understanding of POTS as a post-COVID-19 sequela, was that both naïve POTS and PASC POTS patients would have essentially identical clinical symptoms. This would provide additional evidence that POTS may be preceded by an immunological stressor, such as a viral infection, and that, somehow, platelets may be an important constituent of the disorder.

## 2 Materials and methods

Our prospective case-control study protocol conforms to the ethical guidelines outlined in the 1975 Declaration of Helsinki, as reflected in *a priori* approval by the Institutional Review Board (IRB) of the University of Toledo Medical Center. A total of 252 participants were recruited to create four (4) distinct groups; we had two groups of POTS patients, one group diagnosed prior to the COVID-19 pandemic, and a second group who had developed dysautonomia following a COVID-19 infection (PACS) and subsequently diagnosed with POTS. All POTS patients included in this study had been diagnosed based on history, physical examination, and tilt table test performed in the fasting state. All had a 6-month or greater history of orthostatic intolerance manifested by orthostatic tachycardia with a heart rate increase of at least 30 bpm (or a rate exceeding 120 bpm) observed during the first 10 min of upright posture without orthostatic hypotension, weakness, light-headedness, fatigue, and near syncope (6). All subjects were required to sign our IRB-approved informed consent form.

We categorized patients diagnosed with POTS prior to the pandemic as Naïve POTS (*n* = 70) and those with PACS as POTS post-COVID-19 (*n* = 67). For each group, we attempted to recruit volunteers who matched both age and sex. To prevent confounding variables, patients were excluded from recruitment if taking a SSRI, which can deplete the dense granule of serotonin (43) for therapeutic management of their condition, or had reported having an autoimmune disorder. We recruited two control groups: one naïve to COVID-19 infections (“naïve” controls, *n* = 62) and another group comprising patients who had experienced an infection but recovered healthily (“recovered” controls, *n* = 53). Exclusion criteria for controls were applied after the time of venipuncture. Sixteen individuals were excluded from the naïve control group after ELISA screening for COVID-19 nuclear protein data was obtained, indicating that they had experienced an asymptomatic infection. In addition, exclusion criteria for control groups included a self-reported inflammatory condition (i.e., Hashimoto's disease, inflammatory bowel disease, other autoimmune disorder), taking a SSRI medication, or indications of abnormal bleeding. Naïve controls were not excluded if they had been vaccinated for COVID-19, although several reports have described how the immunologic stressors of both the HPV and COVID-19 vaccinations can induce POTS (25, 44, 45). We also measured antibodies against Epstein–Barr virus infection (EBV), as reactivation of EBV has been reported in PACS (46–49). All volunteers were required to provide peripheral blood samples to determine a complete blood count (CBC), detect SARS-CoV-2 infection, EBV reactivation, and assess their platelet dense granule number.

### 2.1 POTS diagnosis

Tilt table testing utilized a 70° baseline upright tilt for 30 min, during which time heart rate and blood pressure were continuously monitored (50). The test ended if symptomatic hypotension and bradycardia occurred, reproducing the patient's symptoms. If the patient did not experience symptoms, an intravenous infusion of isoproterenol was initiated in the supine position with a dose sufficient to raise the heart rate to 20%−25% above the resting value. Subsequently, upright tilt was repeated for 15 min. Patients and control participants who were on chronic antihypertensive, diuretic, anti-cholinergic, or antidepressant medications, and those with diabetic neuropathy or multisystem disease of any etiology were excluded from recruitment for this study. Each patient underwent a thorough history and physical examination as well as detailed blood chemistry analysis and thyroid profile analysis.

### 2.2 Demographic collections

All subjects, following informed consent, completed four different survey questionnaires, including (1) a modified bleeding assessment tool (BAT) designed by the Scientific and Standardization Committee on Platelet Physiology of the International Society on Thrombosis and Haemostasis to obtain an objective bleeding tendency score that can indicate potential platelet dysfunction (51). Our BAT was modified by changing pronouns only (i.e., “I bruise easily” rather than “do you bruise easily?”) so that participants could complete it themselves, in contrast to having a health professional ask questions on the form. We did not validate the modifications, as the questionnaire context remained unchanged, except for the use of personal pronouns or possessive adjectives (i.e., “my”) for self-assessment. A mean BAT score of 5 or more for women and 3 or more for men is indicative of a platelet function disorder (52, 53). (2) To assess the potential for heavy menstrual bleeding, a common clinical symptom seen in women with bleeding of unknown etiology, we used a pictorial chart to discriminate between menorrhagia and normal menstrual blood loss. A score of 185 or more is predictive of abnormal menstrual bleeding (54, 55). (3) A tool to assess an objective POTS severity of symptoms was used [Composite Autonomic Symptom Score-31 (COMPASS-31)] (56). The COMPASS-31 questionnaire enables the calculation of an objective score from 0 to 100, considering six different categories: orthostatic intolerance, vasomotor, secretomotor, gastrointestinal, bladder, and pupillomotor functions. A COMPASS-31 score >20 is considered indicative of moderate to severe autonomic dysfunction (56, 57). (4) Finally, we modified an assessment tool developed at the University of Vanderbilt Heart Institute to measure a relative symptom score for associated clinical co-morbidities in POTS (58). A total of 149 symptoms/conditions were included, such as dizziness, fatigue, and migraine headache, with each symptom assigned a value of 1. This allowed us to determine an objective “clinical symptom score” for each volunteer.

### 2.3 Platelet preparation

**Figure 1:**
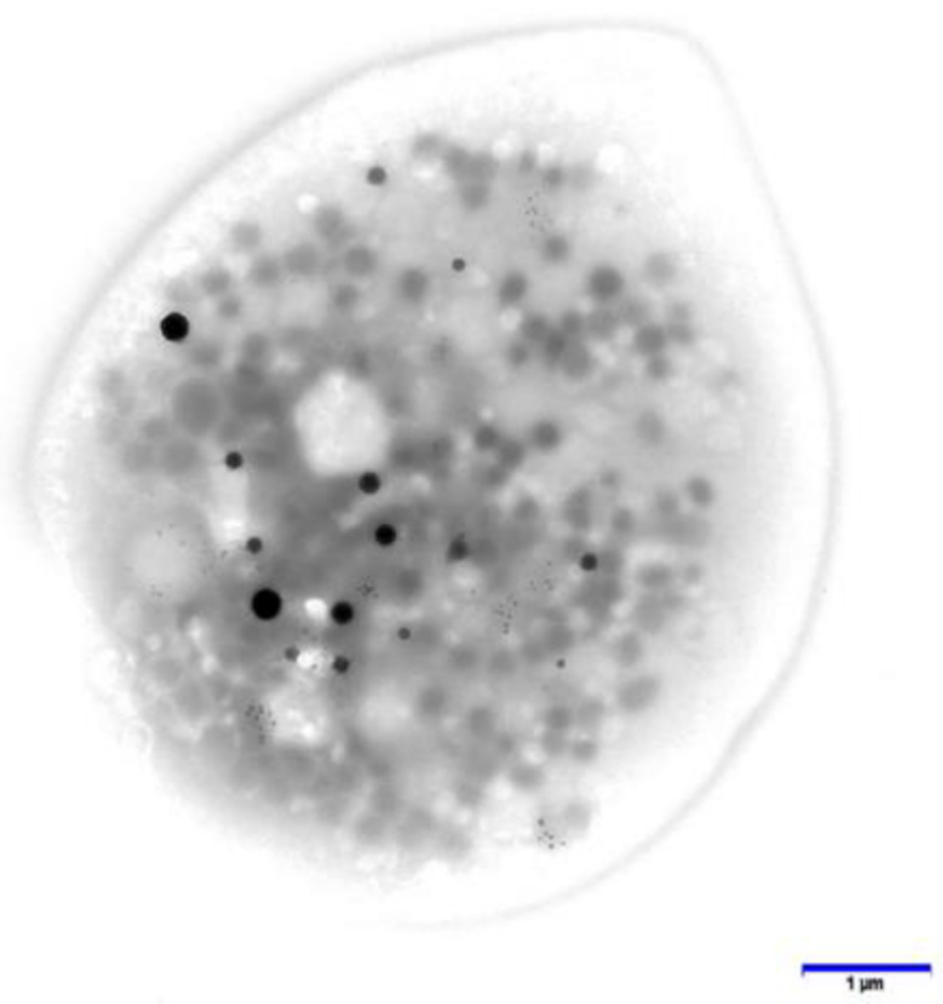
This image is an entire platelet obtained from platelet-rich plasma that had settled upon an electron microscopy support film, washed, and air-dried without fixation. Dense granules are readily apparent due to the opacity of calcium, one of the constituents stored within delta granules. Bar = 1 μm. Original magnification: 10,000×.

Platelet-rich plasma (PRP) was obtained from whole blood by centrifugation at room temperature for 15 min at 200 g. Electron microscopy-coated copper grids used for platelet support were washed with deionized water after PRP incubation and air-dried as described by White (59). A FEI Tecnai G2 Spirit BioTwin transmission electron microscope (Hillsboro, OR, USA) was used to determine an average number of dense granules per platelet (DG/PL). Figure 1 represents a whole-mounted air-dried platelet as viewed by electron microscopy. Dense granules are readily apparent due to contrast with the platelet cytosol as calcium ions are stored in the same organelle that contains adenine nucleotides (mostly ADP), serotonin, and pyrophosphates. Previous studies from our laboratory have established a mean normal value of 4.60 + 0.47 DG/PL(SE), consistent with the established literature (60).

### 2.4. ELISA assays: viral protein assessments

We assessed both COVID-19 and EBV antibodies to evaluate the exposure to further, or state of activity, of these viruses in participants (2, 9, 15, 61, 62). We purchased ELISA kits from EU-ROIMMUN US, Mountain Lakes, NJ, USA, to measure immunoglobulin concentrations to COVID-19 nuclear (catalog# KTR1035) and spike proteins (catalog # KTR1034). For EBV, ELISA kits were purchased from Serion Diagnostics, Wurzburg, GmbH, Germany, to measure antibody titers for viral capsid antigens immunoglobulins M and G [VCAs; VCA Immunoglobulin M (IgM) (catalog # ESR1361M) and VCA IgG (catalog # ESR1361G)], the early antigens immunoglobulin G [EA IgG (catalog # ESR1363G)], and Epstein–Barr Nuclear Antigen Immunoglobulin G [EBNA IgG (catalog # ESR1362G)]. These immunoglobulins enable the determination of whether an individual has had an Epstein–Barr virus infection in the past or if they may have an active or recently active infection. Table 1 (63) is commonly used to assess EBV antibody positivity/negativity and interpretation; the table can be found easily on numerous web pages when searching the internet.

**Table 1:** Serologic profiles of the EBV antibody tests.

**VCA IgM**	**VCA IgG**	**EBNA**	**EA**	**Interpretation**
44 U/ml	40 U/ml	20 U/ml	11 U/ml	Cut-off values (> is positive)
Negative	Negative	Negative	Negative	Seronegative – susceptible
Positive	Positive	Negative	Pos/negative	Acute primary infection
Negative/positive	Positive	Negative/positive	Positive	Recent primary infection
Negative	Positive	Positive	Negative/positive	Past infection
Negative	Positive	Positive	Pos/negative	Reactivated infection

### 2.5 Statistical analysis

Unless otherwise stated, data are presented as mean+1 standard error of the mean (SEM). The chi-squared test, Fisher's exact test, one-way analysis of variance (ANOVA), Mann–Whitney rank-sum test, Kruskal–Wallis one-way ANOVA on ranks, and correlation assays were performed using the SAS System (SAS Institute, Inc., Cary, NC, USA) and graphed using SigmaPlot^®^ (Grafiti, LLC, Palo Alto, CA, USA).

## 3 Results

### 3.1 Group demographics

Our study recruited both men and women; the naïve controls were 73% women, naïve POTS were 91% women, COVID-19 recovered controls were 79% women, and POTS post-COVID-19 were 96% women. This is consistent with the established literature that POTS is a disorder of young premenopausal Caucasian women (28, 64). There were no statistically significant differences among the four study groups regarding complete blood cell counts. All questionnaires that were utilized demonstrated significant differences between control groups and POTS patients. Data collected using our questionnaires provided objective scores for BAT to assess bleeding severity, the menses assessment to detect abnormal menses, the COMPASS-31 to assess the severity of dysautonomia, and an objective clinical symptom measurement. All questionnaire results correlated significantly with platelet dense granule numbers for each group (Table 2).

**Table 2:** Volunteer demographics and questionnaire scores used to assess clinical symptoms in control and POTS groups.

**Assay**	**Naïve controls**	**Naïve POTS**	**COVID-19 recovered**	**POTS post-COVID-19**
Age	34.7 ± 15.3	35.6 ± 13.3	33.7 ± 13.7	38.9 ± 17.4
Sex	73% women	91% women	79% women	96% women
COMPASS-31	14.4 ± 0.9	39.4 ± 1.6^**^	12.6 ± 0.2	33.3 ± 1.8^**^
Bleeding	0.8 ± 0.3	5.1 ± 0.6^**^	0.9 ± 0.3	5.8 ± 0.7^**^
Menses	101.9 ± 15.7	296.6 ± 43.5^**^	109.7 ± 28.1	246.5 ± 53.4^**^
Symptoms score	10.4 ± 1.2	46.9 ± 2.5 ^**^	10.5 ± 1.2	44.7 ± 2.4 ^**^
Platelet dense granule number	4.33 ± 0.61	2.52 ± 0.93^**^	4.19 ± 1.0	2.44 ± 0.94^**^

^**^p < 0.001 for both POTS groups compared to controls.

**Figure 2:**
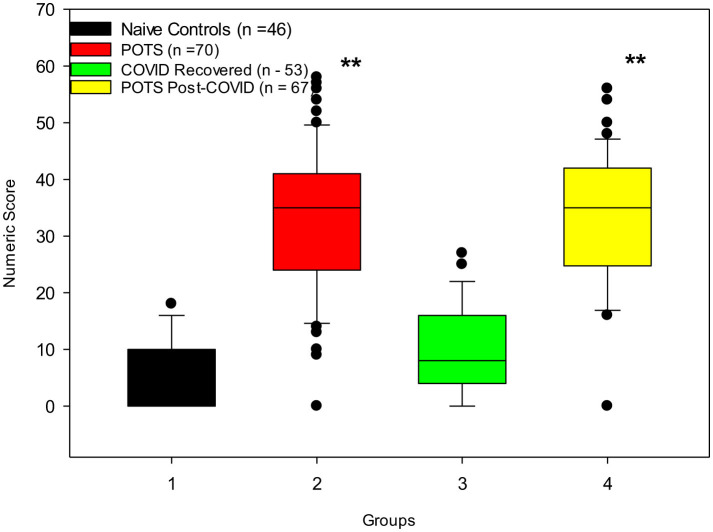
Results of COMPASS-31 scores for control groups (*n* = 99) in comparison to POTS patients (*n* = 137) are significantly different (^**^ *p* < 0.001).

The COMPASS-31 scores are in line with previously published studies (20, 65). Our naïve and COVID-19 recovered healthy control groups had similar low COMPASS-31 scores (< 20, the normal cut-off score) in contrast with naïve POTS (score = 39.4 ± 1.6, *p* < 0.001) and POTS post-COVID-19 (score = 33.3 ± 1.8, *p* < 0.001) patients (Table 2; Figure 2).

Although the BAT was not designed for self-assessment, we found our modified version serviceable. The mean BAT scores for our control groups were normal (scores = 1), whereas our POTS groups had higher bleeding scores (scores = 5+). Regardless of being naïve to COVID-19 infection or having PACS, our POTS patients had BAT scores suggestive of increased bleeding tendencies.

**Figure 3:**
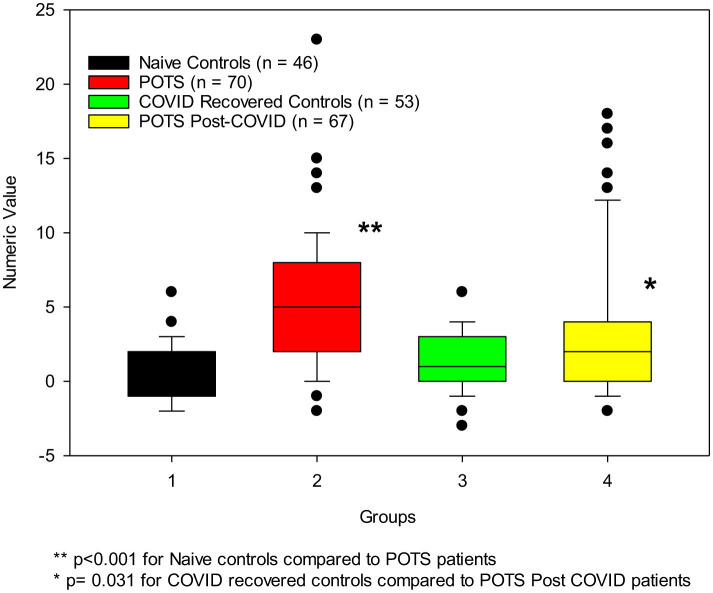
POTS patients (*n* = 137) have increased bleeding tendencies compared to the control groups (*n* = 99).

The results of our menstrual bleeding assessment correlated with the BAT results. Both control groups had normal menses scores < 185, whereas both POTS groups had abnormal menses scores, with naïve POTS patients having a mean of 296.6 ± 43.5 and POTS post-COVID-19 patients having a mean score of 246.5 ± 53.4 (Table 2; Figure 3).

### 3.2 Electron microscopy

**Figure 4:**
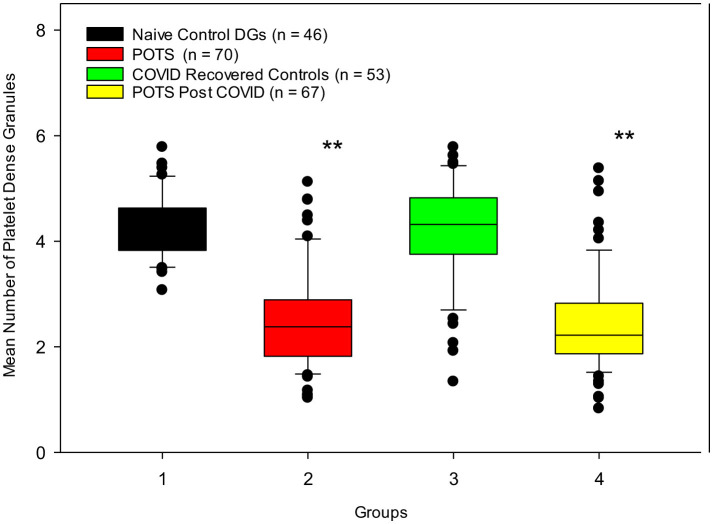
Control subjects who were naïve to COVID-19 infection and those who contracted COVID-19 infection but recovered healthily have normal numbers of dense granules, in contrast to POTS patients diagnosed prior to the pandemic and patients who developed POTS post-COVID-19 infection. ^**^ *p* < 0.001 for both POTS groups vs. control groups.

Assessments of platelet whole mounts also revealed significant differences between our control and POTS groups. Our naïve control group and our COVID-19 recovered healthy group had means of 4.33 ± 0.6 DG/PL and 4.19 ±1.0 DG/PL, respectively, in contrast to our POTS groups. Naïve to COVID-19, POTS patients, and PACS POTS patients were significantly deficient in platelet dense granules, consistent with δ-SPD. Naïve POTS patients were found to have a mean of 2.52 ± 0.9 DG/PL, and POTS post-COVID-19 patients had a mean of 2.44 ± 0.9 DG/PL (Figure 4). Our cut-off value for diagnosing δ-SPD is 3.68 DG/PL, though other laboratories have reported other ranges (60). The use of both a Mann–Whitney rank-sum test and a Kruskal–Wallis one-way analysis of variance on ranks determined significant differences between control groups and POTS patients (*p* < 0.001). POTS. These results have validated our earlier publication that most POTS patients have δ-SPD (11). Not only have we validated our previous study, but we now demonstrate that individuals who developed POTS post-COVID-19 also have a near-identical δ-SPD.

### 3.3 SARS-CoV-2 viral protein assessments

We detected nuclear capsid antibodies against the SARS-CoV-2 virus in volunteers who reported having been infected (Table 3). Our cut-off value for a positive COVID-19 infection was 5.0 U/mL. Volunteers who had been infected with SARS-CoV-2 in the study had a mean anti-SARS-CoV-2 nuclear capsid titer ~3-fold higher than both controls and POTS patients naïve to the virus. Almost all volunteers in all four groups had been vaccinated against COVID-19 when they were recruited, indicated by elevated antibody titers against the SARS-CoV-2 viral spike protein (Table 3).

**Table 3:** Baseline characteristics and comorbidities in patients with postural orthostatic tachycardia syndrome.

**Demographic/ symptom**	**POTS (2016^15^)**	**Naïve POTS**	**POTS post-COVID-19**
Women	96.1%	91%	96%
Fatigue	91.3%	93.2%	92.2%
Easy bruising	71.3%	79.7%	90.5%
Migraine	64.4%	61.1%	81.3%
Irritable bowel syndrome	49.7%	37.3%	59.4%
Cognitive issues	37.5%	69.5%	62.5%
Depression	33.1%	38.9%	34.4%
Epistaxis	33.1%	16.9%	23.4%
Heavy menstrual bleeding	32.1%	38.5%	42.1%
Raynaud's Syndrome	31.7%	32.2%	29.7%
Anxiety	29.8%	39%	50%
Anemia	22.7%	25.4%	25.1%

Of unknown significance is the elevation of spike protein antibodies in the COVID-19 recovered healthy group, which is twice as high as that found in POTS PACS patients. Individuals who had been infected with the virus had higher spike protein antibody titers than those who had only received the vaccination. Interestingly, we detected the anti-nucleocapsid IgG in 11 of the control volunteers who claimed that they had never been infected; these individuals were excluded from the naïve control group. We also evaluated EBV antibodies to determine the potential that POTS post-COVID-19 might be due to a reactivation of EBV, as has been suggested in the literature. As defined in Table 1, criteria to determine a recent or reactivated EBV infection can be determined by using four different antibody titer assays, including IgM and IgG antibodies against the viral capsid antigen (VCA), IgG against the nuclear antigen 1 (EBNA1), and IgG against a complex of early antigens (EA). We did not observe evidence that a recent EBV infection or reactivation had occurred in any of our test subjects. Curiously, both POTS groups had slight evaluations of EBV VCA IgM, but these were well below the positive cut-off values (Table 4).

**Table 4:** Comparison of viral antibody titers between study groups.

**Viral antibody**	**Naïve control**	**Naïve POTS**	**COVID-19-recovered control**	**POTS post-COVID-19**	**Units**
SARS-CoV-2 nuclear capsid IgG	3.62	3.59	17.45^**^	10.86^**^	U/ml
SARS-CoV-2 Spike Protein IgG	335.87	462.71	1,337.75^**^	691.39^**^	U/ml
EBV VCA IgG	117.14	119.29	85.40	105.78	U/ml
EBV VCA IgM	2.93	3.69	2.24	3.90	U/ml
EBV EBNA1 IgG	17.80	24.86	18.50	22.38	U/ml
EBV EA IgG	5.65	7.67	7.50	12.16	U/ml

SARS nuclear capsid IgG > 5.0 = COVID-19 infection.

^**^p < 0.001 for COVID-19 naïve groups vs. infected subjects.

### 3.4 Other common symptoms

A variety of additional symptoms for both our naïve POTS and our post-COVID-19 POTS groups were also noted in their medical history, similar to what we have previously reported (11). The most frequently reported complaints were fatigue, palpitations, easy bruising, irritable bowel syndrome/gastritis, and cognitive issues; for example, these were determined using a modified questionnaire established at Vanderbilt University (58). There were significant differences in reported clinical symptoms between our control patients and our POTS patients (Table 2). The control groups had clinical symptom scores of ~10, whereas both POTS groups had a symptom score of ~45. Comparisons between the symptoms we reported in 2016 and those in our current study are presented in Table 3. Our results are similar to numerous reports in the current literature (20, 66–68).

## 4 Discussion

Orthostatic intolerance (OI) has been reported to affect an estimated 500,000 people in the United States in 1999; partial dysautonomia and hyperadrenergic orthostatic intolerance are also referred to as postural orthostatic tachycardia syndrome (POTS) or sympathetic orthostatic intolerance (69). The etiology of POTS has various proposed causes; many studies have reported preceding viral symptoms (8–10), while others have suspected low levels of serotonin (11–13). Additionally, some of the studies describe the presence of autoantibodies in POTS (15–18). Many clinical morbidities are truly related to low levels of serotonin, and descriptions of POTS patients with circulating G-protein coupled receptor autoantibodies have been reported (21, 24, 27–29, 58, 64, 70, 71). The disorder is often misdiagnosed as chronic anxiety or a panic disorder because the autonomic failure in these patients is not severe, and the variety of clinical symptoms, including fatigue, light-headedness, nausea, headache, near syncope, and exercise intolerance, is subtle (72, 73). Furthermore, POTS may be idiopathic and unrelated to other diseases, with most affected patients categorized with a partial dysautonomic condition appearing to be related to mild peripheral autonomic neuropathy (72, 73).

The same symptoms are now common in PACS, and many of these patients are being diagnosed with neurological or psychiatric problems, just as POTS patients were before the pandemic (74–77). Postural orthostatic tachycardia syndrome is now reported to affect one to three million, with various reports suggesting 0.2% of the population to as many as 170 per 100,000, with additional untold millions more. At least 65 million individuals are estimated to have PACS, and of these, up to 79% are reported to meet international criteria for POTS (21, 24, 58, 64, 70, 71). The current post-pandemic literature on PACS, in relation to POTS, recognizes the association between orthostatic intolerance and a preceding immunologic stressor, such as a virus or vaccine.

The majority of POTS patients require numerous visits to several physicians of different disciplines to be accurately diagnosed due to the multitude of conflicting clinical symptoms (78–80). Hypotheses regarding pathophysiology include abnormally increased sympathetic activity, sympathetic denervation leading to hypovolemia and reflex tachycardia, and autoimmunity to G-protein coupled receptors (2, 3, 79, 81, 82). The autoimmunity may occur via a process called molecular mimicry (30, 31). Molecular mimicry has also been postulated as a mechanism by which viruses evade the immune system by mimicking host proteins, but results in the production of cross-reacting autoantibodies (32). Other studies have postulated that COVID-19 causes a number of neuropathology-related symptoms through an indirect means of upregulating the immune system via molecular mimicking proteins in the vagal nuclei and ganglia (33, 34). Observations reported since the pandemic of a high percentage of PACS patients developing dysautonomia and reports that COVID-19 vaccination induces POTS support the hypothesis that the etiology of POTS involves an immunologic stressor (23–25, 83, 84). Before the pandemic, occasional reports had discussed common associations with Ehlers–Danlos syndrome, low serotonin levels, and potential preceding EBV infections.

More than 90% of our patients reported fatigue (Table 3). The most common bleeding symptom for both POTS groups was easy bruising, but frequent nose bleeds and heavy menstrual bleeding were also reported. The results of our electron microscopy assay of platelet dense granules for all four groups correlate well with our BAT and menses scores, as well as the clinical symptoms commonly associated with platelet dysfunction disorders, including easy bruising, epistaxis, and heavy menstrual bleeding, which are seen in our POTS patients. Our observation of mucocutaneous bleeding in these patients is not as severe as seen in other coagulation system anomalies, typically needed to cause significant bleeding. Most publications of PACS that describe coagulopathies report on thrombosis. Still, one recent study reports “that hemorrhage and risk of hemorrhage are not necessarily an infrequent finding in COVID-19, albeit most probably associated with contributing factors” (85, 86). This report provides support to our findings that both COVID-19-induced POTS and POTS naïve to COVID-19 may be associated with platelet dysfunction, specifically platelet δ-SPD. Both of our control groups were found to have normal numbers of dense granules per platelet.

Notably, we observed that study participants who had been infected with COVID-19 exhibited an approximate 2–4-fold increase in viral spike protein IgG antibody titers compared to controls. As stated previously, with few exceptions, all our volunteers had been vaccinated against COVID-19 at the time of enrollment and venipuncture. We did not consider the evaluation of different manufacturers' vaccines. It is most interesting to observe a nearly two-fold increase in both nuclear capsid IgG and spike protein IgG antibodies in the recovered healthy control groups compared to the POTS post-COVID-19 group. Could this imply that some individuals infected with COVID-19 have a reduced innate and/or adaptive immune response to the virus compared to the majority of people, and therefore are more susceptible to lingering effects or continued chronic infections that subsequently lead to PACS? This question should be further evaluated; why do some people develop PACS and dysautonomia, whereas most infected patients recover fully from COVID-19? Evaluating any difference in viral antibody titers, specifically in those infected with SARS-CoV-2, for comparison with clinical symptoms or variances in platelet dense granule numbers, was not within the scope of our project. These assays were utilized to detect asymptomatic COVID-19 infections as an exclusion criterion for our naïve control group.

We report a significant association with platelet δ-SPD, a type of platelet dysfunction disorder, in POTS patients naïve to COVID-19 infection and in PASC patients diagnosed with POTS (*p* < 0.001 compared to respective control groups). We have validated our previous observation that most POTS patients have platelet δ-SPD. We also demonstrate with statistical significance that COVID-19 long-haulers diagnosed with POTS have essentially identical demographic profiles to COVID-19 naïve patients. The data generated by the use of our questionnaires, including our two bleeding assessment tools, correlate with our platelet dense granule assessment results. Platelet dysfunction disorders such as von Willebrand disease and platelet δ-SPD usually manifest with common symptoms, including easy bruising, frequent nose bleeds, heavy menstrual bleeding for women, excessive bleeding of the gums with brushing, and excessive bleeding with surgery or trauma. Platelet dense granule deficiency is thought to have an autosomal dominant inheritance pattern, but it is also known to be acquired. We postulate that acquired δ-SPD is the result of a chronic inflammatory state induced by an immunological stressor, which could potentially explain the occurrence of δ-SPD in our post-COVID-19 POTS patients. Interestingly, one drug that has been shown to reduce bleeding symptoms related to δ-SPD (Desmopressin) has also been reported to reduce symptoms in POTS (87).

Is our observation that patients diagnosed with POTS have platelet δ-SPD important? It is possible that this may be a factor related to some of the symptoms reported by some POTS patients. Many of their mild bleeding symptoms are suggestive of an underlying platelet dysfunction condition. It is unknown whether or not δ-SPD is a risk factor for the development of POTS. It is possible that δ-SPD is an acquired condition, brought on by an immunological stressor. This is a challenging problem to address, but recent reports of microthrombi found in the blood of PACS patients implicate platelet activation as a result of COVID-19 infection, and may be a factor in some of the symptoms reported by some POTS patients (86, 88, 89).

An additional limitation of our study is that we did not assess serotonin concentrations contained in platelet dense granules. We previously reported that POTS patients have low levels of serotonin, stored in platelet dense granules, when compared to controls (11, 90). The observation of low serotonin in POTS correlates with many symptoms reported by POTS patients. A further limitation of this project is the lack of platelet dense granule data prior to the development of POTS, which hinders consideration of whether δ-SPD is a risk factor for the development of POTS or if it is an acquired condition. We did not utilize PCR to detect EBV for reactivation in this study. For patients with deficient immune systems, in some respects, assessment for EBV infection/reactivation via EBV antibody testing may not be the optimal method for assessing this infection. Finally, we did not assess cohorts for hypermobility spectrum disorders or hypermobile Ehlers–Danlos syndromes that are known to be associated with abnormal bleeding. As these conditions are common comorbidities in POTS, the δ-SPD we report in POTS patients, in contrast to our control cohorts, might be related to these connective tissue disorders rather than POTS.

## 5 Conclusion

We have validated our previously reported data, which indicates that most POTS patients have a platelet delta granule storage pool deficiency. We do not propose that platelet dense granule deficiency is the etiology of POTS; however, it appears that the condition is somehow associated with POTS. Our observation correlates well with clinical symptoms assessed by questionnaires to determine bleeding tendencies, creating an objective score for comparison with control groups. We have also found that patients diagnosed with POTS post-COVID-19 infection (long haulers/PACS patients) have essentially identical demographics when compared to patients diagnosed with POTS prior to the pandemic. What has not been defined is the association between δ-SPD and POTS. Further research is needed to evaluate whether δ-SPD is a risk factor for the development of POTS or an acquired state, related to an inflammatory response to a viral infection such as COVID-19. Our future research plans include reassessing POTS patients recruited for this study to evaluate any improvement in symptoms and, if present, to investigate potential normalization of their platelet dense granule numbers.

## Data Availability

The original contributions presented in the study are included in the article/Supplementary material, further inquiries can be directed to the corresponding author.
