# Supplementary material for: Postural orthostatic tachycardia syndrome in post-COVID-19 long-hauler patients is associated with platelet storage pool deficiency
Source: Front Med (Lausanne). 2025 Sep 11;12:1560120. doi: 10.3389/fmed.2025.1560120 (PMC12460298; doi:10.3389/fmed.2025.1560120)
Supplement: Supplementary file 1 [file Table_1.docx]

Supplemental Table 1.

**Complete Blood Cell Count of Patients Diagnosed with Postural Orthostatic Tachycardia Syndrome and Control Subjects**

| **Category** | **COVID Naïve POTS**  **(n = 70)** | **POTS Post- COVID**  **(n = 67)** | **COVID Naïve Controls**  **(n = 46)** | **COVID Controls**  **(n = 53)** | **Unit** |
| --- | --- | --- | --- | --- | --- |
| RBC | 3.9 ± 0.4 | 3.9 ± 0.4 | 3.9 ±0.5 | 3.9 ±0.4 | 10^6^/µl |
| WBC | 6.5 ± 2.3 | 6.3 ± 1.6 | 5.6 ± 1.4 | 5.9 ± 1.1 | 10^6^/µl |
| NE % | 59.5 ±10.3 | 61.6 ± 8.4 | 57.9 ± 7.6 | 59.2 ± 6.8 | % |
| LY % | 29.9 ± 8.8 | 28.1 ± 7.7 | 30.1 ± 6.9 | 30.2 ± 6.1 | % |
| MO % | 8.3 ± 8.4 | 8.2 ± 6.6 | 8.2 ± 1.9 | 10.2 ± 17.1 | % |
| EO % | 2.2 ± 1.9 | 3.1 ± 6.1 | 2.3 ± 1.9 | 2.7 ± 1.9 | % |
| BA % | 0.6 ± 0.4 | 0.7 ± 0.3 | 0.7 ± 0.3 | 0.7 ± 0.3 | % |
| HGB | 11.4 ±1.1 | 11.6 ± 1.1 | 11.4 ± 1.4 | 11.3 ± 3.7 | g/dL |
| HCT | 37.1 ± 3.2 | 37.8 ± 2.9 | 36.3 ± 1.4 | 36.3 ± 1.4 | % |
| MCV | 95.5 ± 5.0 | 96.1 ± 4.2 | 93.3 ± 6.4 | 93.7 ± 4.4 | fL |
| MCH | 29.4 ± 1.7 | 29.6 ± 0.6 | 29.3 ± 2.5 | 29.4 ± 2.1 | pg |
| MCHC | 30.6 ± 2.1 | 30.9 ± 1.3 | 31.5 ± 1.2 | 31.2 ± 1.1 | % |
| RDW | 13.7 ± 3.3 | 13.9 ±4.9 | 12.9 ± 1.3 | 13.7 ± 4.4 | % |
| PLT | 201.8 ± 59 | 218.5 ± 53 | 188.5 ± 52 | 192.5 ± 42 | 10^6^/µl |
| MPV | 10.0 ± 0.8 | 9.7 ± 0.6 | 9.8 ± 0.7 | 9.9 ± 0.8 | fL |
